# Availability of Observational Pain Assessment Tools in Hospitalized Patients with Osteoporotic Vertebral Fractures

**DOI:** 10.3390/medicina60081217

**Published:** 2024-07-27

**Authors:** Youhei Yoshimi, Takanori Matsuura, Kazuaki Miyazato, Shiho Takahashi, Nami Tanaka, Hanae Morinaga, Asuka Hayata, Minami Onishi, Yousuke Nagano, Hideo Ohnishi

**Affiliations:** 1 Department of Orthopedics, Moji Medical Center, Moji-ku, Kitakyushu 801-8502, Japan; qqna7sbd_1019@yahoo.co.jp (Y.Y.); kmnnmt0528@gmail.com (K.M.);; 2 Department of Orthopedics, School of Medicine, University of Occupational and Environmental Health, Yahatanishi-ku, Kitakyushu 807-8555, Japan; 3 Department of Orthopedics, Nishinomiya Watanabe Hospital, Murokawa-cho, Nishinomiya 662-0863, Japan; 4 Department of Nursing, Moji Medical Center, Moji-ku, Kitakyushu 801-8502, Japan; 5 Department of Rehabilitation, Moji Medical Center, Moji-ku, Kitakyushu 662-0863, Japan

**Keywords:** osteoporotic vertebral fracture, cognitive impairment, Numerical Rating Scale, Abbey-J, Doloplus-2, Mini-Mental State Examination

## Abstract

*Background and Objectives*: Osteoporotic vertebral fractures in older patients cause lower back pain and abnormal posture, resulting in impaired activities of daily living (ADLs). Assessing pain using self-reported assessment tools is difficult, especially in patients with moderate-to-severe cognitive impairment. Recently, observational assessment tools have been used when self-reported ones were difficult to administer. No studies have reported the usefulness of observational assessment tools in patients with acute-phase orthopedic disorders without complication. This study aimed to examine the availability of observational tools for pain assessment in patients with lumbar vertebral fractures. *Materials and Methods*: Patients admitted to our hospital with acute-phase vertebral fractures were enrolled in this prospective observational study. Pain was assessed using Japanese versions of the Abbey pain scale and Doloplus-2 observational assessment tools, and the Numerical Rating Scale, a self-reported assessment tool. To compare the pain assessment tool, we examined whether each tool correlated with ADLs and ambulatory status. ADLs were assessed using the Barthel Index. Ambulatory status was assessed using the Functional Ambulation Categories and the 10-m walking test. *Results*: Similar to the Numerical Rating Scale scores, assessments with the Abbey pain scale and Doloplus-2 showed significant decreases in scores over time. A significant positive correlation was observed between the self-reported and observational assessment tools. Each pain assessment tool was significantly negatively correlated with ADLs and ambulatory status. *Conclusions*: When self-reported assessment with the Numerical Rating Scale is difficult for patients with cognitive impairment, pain can be estimated using the Abbey pain scale and Doloplus-2 observational assessment tools.

## 1. Introduction

Osteoporotic vertebral fractures occur in older adults and result in a functional loss in activities of daily living (ADLs) because of the prolonged lower back pain and the resulting abnormal posture [1,2,3,4,5]. In Japan, recent studies have shown that musculoskeletal disorders due to falls and resultant fractures are one of the main reasons the older adults require nursing care [6]. With an increasing number of older adults requiring nursing insurance, its cost has correspondingly increased, which this has become a social problem in Japan. Thus, preventing severe osteoporosis and low-trauma fractures is also important in terms of health economics [2,7]. The annual incidence of vertebral fractures in Japan is approximately 1558 per 100,000 people (women, 2117; men, 729; women-to-men ratio, 2.90), and in Kure City, Hiroshima Prefecture, Japan, the 2015 population aging rate was reported to be 32.9% [8].

Conservative orthotic treatment is the major treatment approach for fresh osteoporotic vertebral fractures unless they are burst fractures [9,10]. At our hospital, when providing outpatient treatment to a patient is challenging, the patient is hospitalized for treatment, which is aimed primarily at pain control using bracing medication and rehabilitation to prevent muscle atrophy. The period of hospitalization may be lengthy, depending on the pathological condition, level of physical activity, and social background (e.g., living alone) of the patient. One of the criteria for discharge from the hospital is reduced lower back pain and the patient’s ability to walk independently. Pain intensity is mainly evaluated by the Numerical Rating Scale (NRS), Visual Analog Scale (VAS), Verbal Rating Scale (VRS), and Faces Pain Rating Scale (FPS). Evaluation of pain during hospitalization is generally measured by a self-assessment method using a NRS or a VAS. However, pain is difficult to assess in cognitively impaired patients with osteoporotic vertebral fractures, and the validity of pain assessments may be doubtful [11,12,13,14,15]. The International Association for the Study of Pain recommends verbal communication tools, such as self-report assessments, in patients with early cognitive memory decline [4], but these tools are difficult to use for assessing patients with advanced dementia [16,17]. In recent years, the use of observational assessment tools has been proposed as an alternative approach for patients where self-reported pain assessment is difficult to perform [18,19,20,21,22,23]. No studies have reported on the usefulness of observational assessment tools for patients with acute-phase orthopedic disorders without comorbidities. Furthermore, the most appropriate observational assessment tool to use is unknown [24].

Our ward is one of the primary super-aging districts in Japan. In March 2020, 36.5% of the population was aged 65 years or older (94,355 individuals; men-to-women ratio: 43,205:51,150). Many patients who have been hospitalized experience cognitive decline. To enable adequate pain relief and rehabilitation of osteoporotic vertebral fractures, optimizing pain assessment tools for patients with cognitive decline is an important issue in acute medical care [12,14,15].

The present study aimed to prospectively evaluate the validity of observational assessment tools. To this end, pain in patients who were admitted to our hospital with acute-phase vertebral fractures was assessed using the Japanese version of the Abbey pain scale (Abbey-J) [25,26] as an observational assessment tool, in addition to the NRS, a self-report assessment tool. The scores of each assessment tool were examined for changes and correlations over time after admission. Furthermore, to evaluate their availability, we determined whether each pain assessment tool correlated with ADLs and ambulatory status.

## 2. Materials and Methods

### 2.1. Ethics Approval Statement

This study was approved by the ethical review board of the Medical Center (approval number, 03-02) and was conducted in compliance with the Declaration of Helsinki. After disclosing all study-related information, the patients were given an opportunity to opt-out.

### 2.2. Participants

Moji Ward in Kitakyushu City is one of the main super-aging districts in Japan, with the percentage of the population aged ≥ 65 years estimated at 36.5% in March 2020 (n = 94,355; 43,205 men, 51,150 women). Many of the hospitalized patients experience a decline in their cognitive function. Of the 385 patients who visited our hospital with a chief complaint of lower back pain and were diagnosed with vertebral fractures between April 2018 and March 2020, 88 patients aged ≥ 65 years were admitted to our hospital (Moji Medical Center). In this study, a total of 35 patients aged 65 years or older who visited our medical center with the main complaint of low back pain before subsequently being hospitalized and diagnosed with a vertebral body fracture between April 2021 and March 2022 were prospectively enrolled. The exclusion criteria were as follows: (1) the presence of complications with vertebral fractures; (2) the individual is confined to bed; and (3) the individual is unable to participate in the rehabilitation programs.

This study does not include patients with burst fractures who required surgery, but only patients who were treated conservatively and followed up. Furthermore, patients with serious complications, such as pneumonia during hospitalization, were excluded.

### 2.3. Assessment

#### 2.3.1. Pain Assessment Using Self-Reported and Observational Assessment Tools

Nurses assessed the pain of the patients at rest and during movement (during bathroom visits) over 10 consecutive days from the day of admission, using the Abbey-J (observational) assessment tools. Then, the nurse assessed the pain of the patients at rest and during movement (during bathroom visits) using the NRS (self-reported) assessment tools on the same day. The NRS is a verbal communication tool in which patients rate pain on an 11-point scale ranging from 0 (no pain) to 10 (worst pain). For the Abbey-J, pain is rated on a scale of 0 to 3 points (a maximum total score of 18 points) on six items reflecting behaviors such as changes during specified movements, vocalizations, and facial expressions. The pain intensity is graded into four grades: no pain (0–2 points), mild pain (3–7 points), moderate pain (8–13 points), or severe pain (14–18 points).

Information on the living conditions of each patient was surveyed, and nurses assessed the pain of each patient twice a week on days 4 and 7 using the Doloplus-2 tool. The Doloplus-2 is an observational tool that was developed for older individuals with chronic pain who are unable to communicate about their pain. The pain intensity was scored from 0 to 3, with higher scores indicating more severe pain. The maximum score was 30 points, with a score of ≥5 points indicating pain.

The assessments were performed by the nurses in charge of the patients on the corresponding days, and the evaluators were not fixed. In consideration of the variability among evaluators, the mean scores on days 1–4, 5–7, and 8–10 were calculated for analyses.

#### 2.3.2. Assessment of Ambulatory Status and ADLs

On admission, the attending physician determined the patient’s ambulatory status before the injury occurred by taking the patient’s history. The patients were then instructed to wear a corset. A physical therapist evaluated the ADLs using the Barthel Index (BI) once a week during the hospital stay. Ambulatory status was evaluated using the functional ambulation categories (FAC) and 10-m walking test results once a week, on days 7, 14, 21, 28, and 35. The BI is a 10-item assessment tool for ADLs that yields a total possible score of 100 points. Based on the BI, a person was classified as being independent, requiring partial assistance, or being totally dependent. The 10 items include eating, moving, grooming, toileting, bathing, walking (moving), going up/downstairs, dressing, defecating, and urinating. The FAC, originally used in patients with stroke, is a clinical assessment index of walking ability based on the amount of assistance required. Walking ability is classified into six categories (categories 1–6) that are based on the observation of movement.

#### 2.3.3. Evaluation of Pain Assessment Tool Scores Using the Mini-Mental State Examination

The Mini-Mental State Examination (MMSE) is a neuropsychological screening test for dementia used to objectively determine which cognitive function is impaired and to what extent. From a possible total score of 30 points, a score of ≤23 points strongly suggests a cognitive decline [27]. The MMSE-J is the authorized Japanese translation published by Nihon Bunka Kagaku-sha under the permission of Psychological Assessment Resources, Inc. Individuals who purchased the MMSE-J test forms provided permission to use them as part of the current research. MMSE scores classify the degree of cognitive decline as normal (score, 24–30 points), mild (score, 18–23 points), or moderate to severe (score, 0–17 points).

### 2.4. Sample Size Determination

Approximately 50 study patients are needed to achieve reasonable accuracy in estimating reliability. However, this number of patients may not be absolute, depending on the design of the study and the test [28]. For this study, we decided on a sample of 30 patients for the period. This is because this study is data from a single institution, and it is focused on inpatients with vertebral fractures, or a single orthopedic condition. Furthermore, the number of patients with vertebral fractures in our hospital over the past two years has been around 40 each year. For our hospital, pain assessment tools have been shown to be useful in assessing pain. In this study, we also considered the inability to assess the NRS when the MMSE is low, so five additional patients were included in the study. In this study, to determine each criterion’s validity, NRS served as the gold standard and was held up against the APS and Doloplus-2 sum scores.

### 2.5. Statistical Analysis

Easy R (EZR) software version 1.60 was used to analyze the data. Univariate analysis was performed to compare the groups using repeated calculations via the Friedman chi-squared test, the analysis of variance (ANOVA), the one-way ANOVA, and the Mann–Whitney *U* test. Considering multicollinearity, Pearson’s and Spearman’s rank correlation coefficient was calculated to describe the associations between the evaluation items. The absolute values found using R were as follows: weak 0.20–0.39, moderate 0.40–0.59, strong 0.60–0.79, and very strong, 0.80–1.00. Significant differences were defined when *p* < 0.05.

## 3. Results

### 3.1. Participant Characteristics

A total of 35 patients (mean age, 84.40 ± 6.65 years [range, 67–95 years]; 31 women) with osteoporotic vertebral fractures were included. Table 1 presents the base characteristics of the patients.

### 3.2. Changes in the Scores of Each Pain Assessment Tool over Time

At first, the NRS tool was difficult to assess in some patients with moderate cognitive decline with MMSE scores of less than 17 (n = 5/6). The NRS, Abbey-J, and Doloplus-2 scores decreased significantly over time after admission (repeated Friedman chi-squared followed by the Bonferroni test).

**Figure 1:**
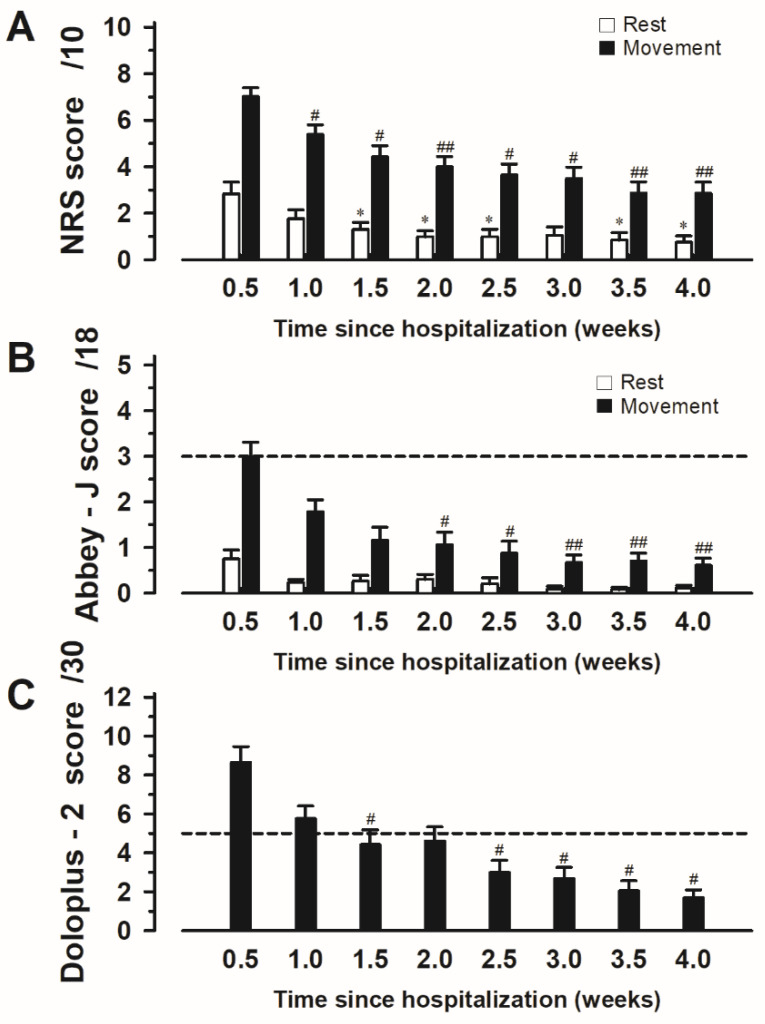
Changes in the scores for each pain assessment tool in hospitalized patients with vertebral fractures. Assessments of the (**A**) NRS, (**B**) Japanese version of the Abbey pain scale (Abbey-J), and (**C**) Doloplus-2 scores are shown. Notes: # *p* < 0.05 and ## *p* < 0.01 compared with 0.5 weeks evaluation (at the beginning of admission) is statistically significant in each scale. * *p* < 0.05 compared with 0.5 weeks evaluation (rest time at the beginning of admission) is statistically significant in each scale. Abbreviations: NRS, Numerical Rating Scale.

Results for the NRS scores during rest were as follows: Friedman chi-squared = 42.516, df = 7, *p* < 0.001; and at 0.5 weeks vs. 1.0 weeks: *p* = 0.167, vs. 1.5 weeks: *p* = 0.034, vs. 2.0 weeks: *p* = 0.049, vs. 2.5 weeks: *p* = 0.035, vs. 3.0 weeks: *p* = 0.059, vs. 3.5 weeks: *p* = 0.031, vs. 4.0 weeks: *p* = 0.020. Results for the NRS scores during movement were as follows: Friedman chi-squared = 80.029, df = 7, *p* < 0.001; and at 0.5 weeks vs. 1.0 week: *p* = 0.044, vs. 1.5 weeks: *p* = 0.004, vs. 2.0 weeks: *p* = 0.006, vs. 2.5 weeks: *p* = 0.015, vs. 3.0 weeks: *p* = 0.010, vs. 3.5 weeks: *p* = 0.002, vs. 4.0 weeks: *p* = 0.004 (Figure 1A).

The Abbey-J scores during rest yielded the following results: Friedman chi-squared = 57.879, df = 7, *p* < 0.001; and at 0.5 weeks vs. 1.0 week: *p* = 0.070, vs. 1.5 weeks: *p* = 0.091, vs. 2.0 weeks: *p* = 0.440, vs. 2.5 weeks: *p* = 0.091, vs. 3.0 weeks: *p* = 0.073, vs. 3.5 weeks: *p* = 0.279, vs. 4.0 weeks: *p* = 0.220. The Abbey-J scores during movement yielded the following results: Friedman chi-squared = 57.879, df = 7, *p* < 0.001; and at 0.5 weeks vs. 1.0 week: *p* = 0.122, vs. 1.5 weeks: *p* = 0.073, vs. 2.0 weeks: *p* = 0.035, vs. 2.5 weeks: *p* = 0.012, vs. 3.0 weeks: *p* = 0.002, vs. 3.5 weeks: *p* = 0.004, vs. 4.0 weeks: *p* = 0.004 (Figure 1B).

Analyses of the Doloplus-2 scores provided the following results: Friedman chi-squared = 56.224, df = 7, *p* < 0.001; and at 0.5 weeks vs. 1.0 week: *p* = 0.364, vs. 1.5 weeks: *p* = 0.020, vs. 2.0 weeks: *p* = 0.071, vs. 2.5 weeks: *p* = 0.028, vs. 3.0 weeks: *p* = 0.031, vs. 3.5 weeks: *p* = 0.013, vs. 4.0 weeks: *p* = 0.020 (Figure 1C).

**Figure 2:**
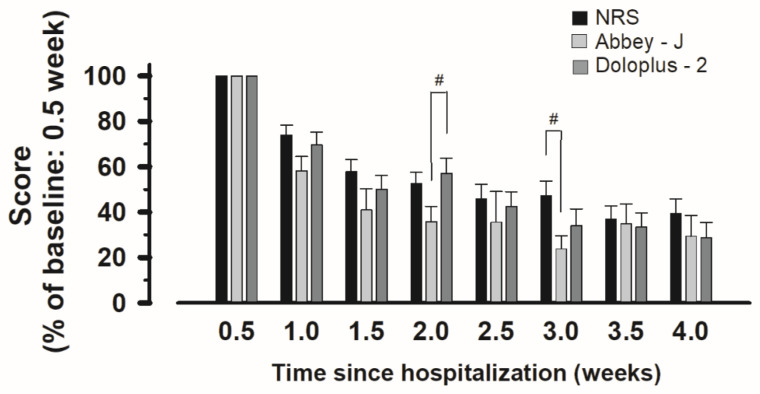
Changes in the scores for each pain assessment tool in hospitalized patients with vertebral fractures. The pain score at admission was assumed as 100. Notes: # *p* < 0.05 compared with NRS, Abbey-J, and Doloplus-2 assessment tools (at the same time after admission) is statistically significant. Abbreviations: NRS, Numerical Rating Scale.

Next, we evaluated the rate of change in scores over the course of hospitalization, with pain at 0.5 weeks after admission for each score set to 100%. At 2.0 weeks and 3.0 weeks post-admission, significant differences in the change of Abbey-J scores during movement and in the NRS or Doloplus-2 scores were observed among the three groups (NRS: 52.54 ± 5.030, Abbey-J: 35.82 ± 6.750, Doloplus-2; 57.14 ± 6.66, one-way ANOVA followed by the Bonferroni, F2,92 = 3.325, *p* = 0.049; Abbey-J compared with Doloplus-2, *p* > 0.05; compared with the others at 2.0 weeks) (NRS: 47.36 ± 6.382, Abbey-J: 23.84 ± 5.700, Doloplus-2; 34.13 ± 7.24, one-way ANOVA followed by the Bonferroni, F2,71 = 3.38, *p* = 0.034; Abbey-J compared with NRS, *p* > 0.05; compared with the others at 3.0 weeks). No significant differences were observed at any other time points (one-way ANOVA at 1.0 week: F2,97 = 2.139, at 1.5 weeks: F2,97 = 1.400, at 2.5 weeks: F2,78 = 0.329, at 3.5 weeks: F2,59 = 0.060, at 4.0 weeks: F2,55 = 0.689, *p* > 0.05; compared with each group) (Figure 2).

Thus, the Abbey-J score showed a significant difference only at 2 or 3 weeks post-admission. Since the Abbey-J is a relatively simple observational assessment tool, adequately evaluating the transition from acute to chronic pain intensity may not be possible.

### 3.3. Correlation between Pain Assessment Tools

At the 2- and 3-week marks of post-admission, the NRS score was significantly positively correlated with both the Abbey-J and Doloplus-2 scores. The correlation coefficients were all moderate to strong (Table 2 blue box).

### 3.4. Changes in ADLs and Ambulatory Status over Time

**Figure 3:**
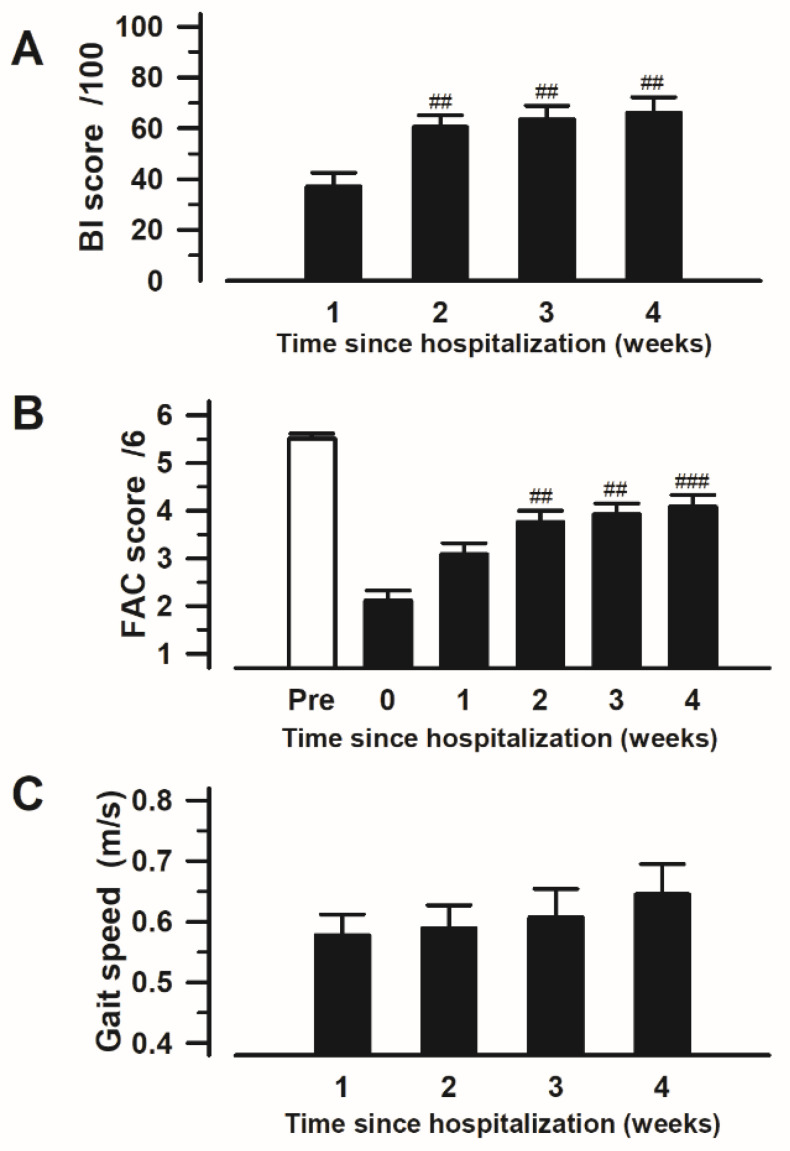
Changes in the scores for activities of daily living and ambulatory status in hospitalized patients with vertebral fractures: (**A**) Barthel Index (BI); (**B**) Functional Ambulation Categories (FAC); and (**C**) 10-m walking test. Notes: ## *p* < 0.01; and ### *p* < 0.001 compared with first evaluation after hospitalization is statistically significant in each scale. Abbreviations: BI, Barthel Index; FAC, Functional Ambulation Categories.

Ambulatory status evaluated using the FAC significantly improved over time after admission. ADLs evaluated using the BI and 10-m walking tests were not significantly different; however, an increasing trend was observed (repeated-measures Friedman chi-squared followed by the Bonferroni test; BI: Friedman chi-squared = 46.577, df = 3, *p* < 0.001; and at 1.0 week vs. 2.0 weeks: *p* = 0.005, vs. 3.0 weeks: *p* = 0.003, vs. 4.0 weeks: *p* = 0.001 (Figure 3A). The results for the FACs were as follows: Friedman chi-squared = 53.671, df = 4, *p* < 0.001; and at 0.5 weeks vs. 1.0 week: *p* = 0.235, vs. 2.0 weeks: *p* = 0.022, vs. 3.0 weeks: *p* = 0.001, vs. 4.0 weeks: *p* = 0.001 (Figure 3B). For the 10-m walking test, the following results were calculated: Friedman chi-squared = 15.058, df = 3, *p* = 0.001; and at 1.0 week vs. 2.0 weeks: *p* = 0.146, vs. 3.0 weeks: *p* = 0.109, vs. 4.0 weeks: *p* = 0.055 (Figure 3C).

### 3.5. Correlation between ADLs and Ambulatory Status

At the 2- and 3-week marks of post-admission, the BI was significantly positively correlated with both the FAC and 10-m walking test results, both of which assess ambulatory ability. The correlation coefficients were all moderate to strong. (Table 2 yellow box).

### 3.6. Correlation of Each Pain Assessment Tool with ADLs and Ambulatory Status

At the 2- and 3-week marks of post-admission, each pain assessment tool showed a relatively negative correlation with the assessment tools for ambulatory status and ADLs. The correlation coefficients were moderate. (Table 2 green box).

### 3.7. Evaluation of Scores Assigned to Items in Each Pain Assessment Tool According to MMSE Scores

**Figure 4:**
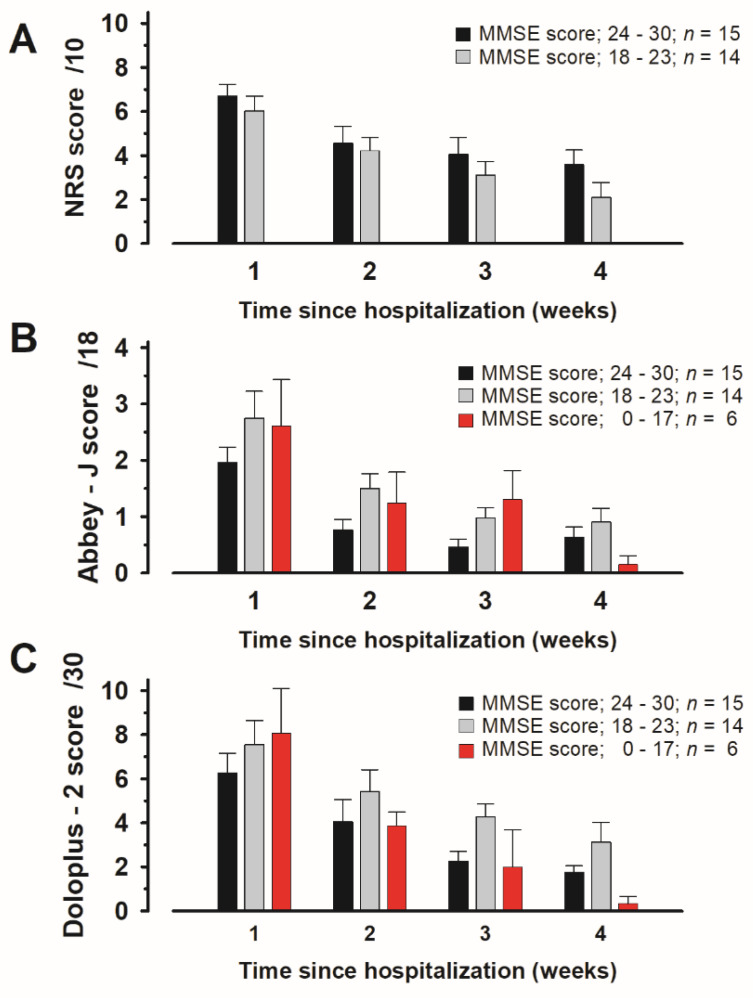
Changes in the scores for each pain assessment tool of hospitalized patients with vertebral fractures according to the degree of cognitive impairment: (**A**) Numerical Rating Scale (NRS); (**B**) Japanese version of the Abbey pain scale (Abbey-J); and (**C**) Doloplus-2. Abbreviations: MMSE, Mini-Mental State Examination.

Each pain assessment tool was used to evaluate the pain of patients with vertebral fractures according to their degree of cognitive impairment. Of the six patients with an MMSE score of ≤17, only one patient was able to assess their pain using the NRS. Pain was difficult to assess in many patients, indicating that the NRS was insufficient for pain assessment. While the NRS assessments at 1.0–4.0 weeks were not significantly different between patients with an MMSE score of 18–23 and those with an MMSE score of ≥24 (at 1.0 week; *p* = 0.895, at 2.0 week; *p* = 0.827, at 3.0 week; *p* = 0.938, at 4.0 week; *p* = 0.568 by Mann–Whitney U followed by the Bonferroni test) (Figure 4A). Furthermore, the scores on the observational assessment tools were not significantly different between patients with an MMSE score of ≤17 and those with an MMSE score of ≥18, and the scores significantly decreased within 4 weeks. However, the Abbey-J and Doloplus-2 assessments at 4 weeks showed decreased tendency among the three MMSE groups; Kruskal-Wallis was followed by the Bonferroni test (Figure 4B,C). The results for the Abbey-J scores during movement at 1.0 week were Kruskal-Wallis chi-squared = 1.093, df = 2, *p* = 0.578; at 2.0 weeks: Kruskal-Wallis chi-squared = 2.207, df = 2, *p* = 0.331; at 3.0 weeks: Kruskal-Wallis chi-squared = 2.563, df = 2, *p* = 0.277 and at 4.0 weeks: Kruskal-Wallis chi-squared = 1.379, df = 2, *p* = 0.501. The results for the Doloplus-2 scores during movement at 1.0 week were Kruskal-Wallis chi-squared = 1.379, df = 2, *p* = 0.501; at 2.0 weeks: Kruskal-Wallis chi-squared = 1.044, df = 2, *p* = 0.593; at 3.0 weeks: Kruskal-Wallis chi-squared = 4.263, df = 2, *p* = 0.118 and at 4.0 weeks: Kruskal-Wallis chi-squared = 4.029, df = 2, *p* = 0.133.

## 4. Discussion

In the present study, all NRS (a self-reported assessment tool), Abbey-J, and Doloplus-2 (observational assessment tools) scores of hospitalized patients with acute-phase vertebral fractures decreased significantly over time after admission. The NRS score positively correlated with both the Abbey-J and Doloplus-2 scores. In addition, each pain assessment tool negatively correlated with ADLs and ambulatory status. These findings verified that observational assessment tools are valid for assessing pain in patients with acute-phase vertebral fractures. However, the change of the Abbey-J score showed a significant difference at 2.0 or 3.0 weeks post-admission compared with the other scales. Since the Abbey-J is a relatively simple observational assessment tool, adequately evaluating the transition from acute to chronic pain intensity may not be possible.

The NRS is an extremely simple self-reported pain assessment tool that is widely used in clinical settings and does not require writing instruments. The pain intensity scales used are typically the NRS, VAS, Verbal Rating Scales (VRS), and faces pain rating scale (FPS) [29] While there are situations wherein the VAS, VRS, or FPS are more appropriate [30,31] there is general consensus that the NRS is more valid and superior than the other scales [32,33,34]. The NRS can be used in patients with mild cognitive impairment, as defined by an MMSE score of ≥18 points. However, for patients with MMSE scores of ≤17 points, NRS scores are reportedly difficult to record [11]. The Abbey-J was developed as an observational tool to assess pain intensity in individuals with dementia. Its use is also relatively simple [35]. In the current study, the Abbey-J scores for lower back pain associated with vertebral fractures were relatively low. One of the characteristics of this study is that pain was classified as mild in several patients. In addition, the pain scores on the Doloplus-2 tended to decrease within a short period after admission. Pain scores evaluated with observational assessment tools may deviate from the actual severity of clinical symptoms (such as lower back pain). However, the knowledge that pain decreases over time may be sufficient for pain assessment.

Of the patients with vertebral fractures examined, the self-reported assessment tool positively correlated with observational assessment tools. Considering that the present study included older patients and those with cognitive impairment, our results demonstrated that the Abbey-J and Doloplus-2 were equivalent to the widely used NRS in the current clinical setting. These observational assessment tools may also be suitable for estimating the pain intensity associated with acute-phase orthopedic disorders that are common in older patients.

The results showed a positive correlation between the BI and FAC in study weeks two and three, indicating that ADLs can be determined by evaluating the ambulatory status in older patients with vertebral fractures, including those with cognitive impairment (Table 2). The negative correlation of each pain assessment tool with ADLs and ambulatory status suggests that the observation of both ambulatory status and ADLs can contribute to pain assessment. The degree of correlation differed somewhat between the self-reported and observational assessment tools in this study; however, observational assessment tools may be used in the future, even for patients for whom assessing pain using the NRS is difficult. Given that a self-reported pain assessment tool such as the NRS is a subjective approach, different pain thresholds among individuals will affect its use. Poor objectivity is one of the limitations of self-reported assessment tools. Thus, pain cannot be objectively assessed in patients for whom self-evaluation of pain is difficult, such as those with cognitive impairment. In this context, assessors’ objective approaches to scoring pain based on patient behavior appear to be easy to perform because the evaluation criteria have been determined [36,37].

When the scores on each pain assessment tool were compared in patients with vertebral fractures according to the degree of cognitive impairment, pain tended to be assessed as milder with the Abbey-J and Doloplus-2 tools than with the NRS tool in patients with cognitive impairment (Figue4 B and C at 4.0 weeks). Determining which tool is superior or inferior based solely on these results is difficult. However, patients with cognitive impairment may not be able to understand, recognize, or express pain itself. Thus, their expression of pain may deviate from their behavior [38]. The availability of the observational assessment tools demonstrated in the current study may have implications in clinical settings because pain intensity can be determined based on behavior. The Abbey and Doloplus-2 tools are appropriate for pain assessment in patients with moderate-to-severe dementia [35,39]. However, evaluators may need to be proficient in observing patient conditions to assess nonverbal communication, such as facial expressions and behaviors, in greater detail. In that sense, the fact that the Abbey-J and Doloplus-2 scores in patients with an MMSE score of ≤17 points and in patients with an MMSE score of ≥18 points at 4 weeks showed decreased tendency may suggest the need for proficient observational skills to be employed when assessing pain. Additionally, considering that observational assessment tools are objective, patients’ pain may have been underestimated.

A limitation of this study is that observational assessment tools may underestimate pain in patients with mild-to-moderate pain. The scoring is also prone to inter-evaluator differences. In observational assessments, pain other than lower back pain may also be simultaneously and inadvertently assessed. Therefore, caution should be exercised when interpreting the results. In particular, patients must be monitored for the onset of complications after admission as factors other than lower back pain may substantially change the scores of the observational assessment tools.

## 5. Conclusions

The self-reported NRS scores in patients who were treated with conservative therapy for acute vertebral fractures at our hospital were significantly positively correlated with observational assessments using the Abbey-J or Doloplus-2 tools. All pain assessment tools were significantly negatively correlated with ADLs and ambulatory status. These results suggest that observational assessments using the Abbey-J or Doloplus-2 assessment tools can be used to estimate pain, even in patients with cognitive impairment where self-reported assessments with the NRS tool are difficult to perform.

## Figures and Tables

**Table 1:** Patient characteristics.

Characteristic	Value
Age (year)	84.4 ± 6.65
Sex; male: female	4(11.4%): 31 (88.6%)
Height (cm)	147.3 ± 7.97
Weight (kg)	46.3 ± 9.46
Body mass index (kg/m^2^)	21.3 ± 4.02
Fracture (number) (multiple fractute included)	T7 (1), T8 (2), T9 (1), T10 (2), T11 (2), T12 (12), L1 (6), L2 (7), L3 (3), L5 (1)

**Table 2:** Correlation of each pain assessment tool with activities of daily living and ambulatory status at weeks 2 and 3 of hospitalization.

2 Weeks	NRS	Abbey	Doloplus-2	BI	FAC
Abbey	0.476 ^###^				
Doloplus-2	0.663 ^###^	0.587 ^###^			
BI	−0.101	−0.542 ^###^	−0.258		
FAC	−0.101	−0.406 ^#^	−0.299	0.796 ^###^	
Gait Speed	−0.461 ^#^	−0.372	−0.537 ^##^	0.576 ^##^	0.608 ^###^
3 Weeks	NRS	Abbey	Doloplus-2	BI	FAC
Abbey	0.608 ^##^				
Doloplus-2	0.484 ^#^	0.585 ^##^			
BI	−0.272	−0.44 ^#^	−0.443 #		
FAC	−0.244	−0.359	−0.371	0.816 ^###^	
Gait Speed	−0.245	−0.386	−0.585 ^###^	0.653 ^###^	0.605 ^##^

Note: Pearson’s and Spearman’s rank correlation coefficient: # *p* < 0.05; ## *p* < 0.01; and ### *p* < 0.001 is statistically significant. Abbreviations: BI, Barthel Index; FAC, Functional Ambulation Categories; NRS, Numerical Rating Scale.

## Data Availability

The data that support the findings of this study are available on request from the corresponding author. The data are not publicly available because of privacy or ethical restrictions.
